# Research note: Effect of a biotechnologically produced *Pleurotus sapidus* mycelium on expression of genes involved in protein synthesis and degradation in breast muscle of broilers

**DOI:** 10.1016/j.psj.2024.104450

**Published:** 2024-10-29

**Authors:** Lea Schäfer, Javier Herrero-Encinas, Martin Rühl, Holger Zorn, Erika Most, Klaus Eder, Robert Ringseis

**Affiliations:** a Institute of Animal Nutrition and Nutrition Physiology, Justus Liebig University Giessen, Giessen, Germany; b ETS Ingenieria Agronómica, Alimentaria y de Biosistemas, Departamento de Pruducción Agraria, Universidad Politécnica de Madrid, Madrid, Spain; c Institute of Food Chemistry and Food Biotechnology, Justus Liebig University Giessen, Giessen, Germany; d Fraunhofer Institute for Molecular Biology and Applied Ecology IME, Giessen, Germany; e Center for Sustainable Food Systems, Justus Liebig University Giessen, Giessen, Germany

**Keywords:** Broilers, Breast muscle, Fungal mycelium, Protein synthesis, Protein degradation

## Abstract

Recently, feeding a fungal mycelium from *Pleurotus sapidus* was found to reduce relative breast muscle weight of broilers. The present study tested the hypothesis that dietary inclusion of *P. sapidus* mycelium modulates the expression of genes involved in protein anabolic and protein catabolic pathways in breast muscle of broilers. The study included 72 male, 1-day-old Cobb 500 broilers which were randomly assigned to three groups fed three different diets containing either 0 (PSA-0), 25 (PSA-25) and 50 (PSA-50) g/kg diet *P. sapidus* mycelium in a three-phase feeding system for 35 days. Within the somatropic axis, the mRNA level of *GHR* was higher and that of *IGF1R* was lower in group PSA-25 than in group PSA-0 (*P* < 0.05). Within the mTOR signaling pathway, the mRNA level of *S6K1* was higher in group PSA-25 than in group PSA-0 (*P* < 0.05). Within muscle growth-related genes, the mRNA level of *MYOG* was lower in groups PSA-25 and PSA-50 than in group PSA-0 (*P* < 0.05). The relative phosphorylation of proteins involved in protein anabolic pathways (S6K1, RPS6, eIF2a, AKT) did not differ across the three groups. The mRNA of most genes involved in molecular pathways of protein degradation and inhibition of protein synthesis, such as the GCN/eIF2a pathway, the ubiquitin-proteasome pathway, and the autophagy-lysosomal pathway, showed no differences across the three groups. Only the mRNA level of *ATG9A* was higher in group PSA-25 compared to group PSA-0 (*P* < 0.05). These observations suggest that a modulation of these signaling pathways is unlikely to explain the reduced relative breast muscle weight in broilers. Nevertheless, future studies are necessary to exclude an effect of feeding *P. sapidus* mycelium on other less prominent pathways affecting skeletal muscle mass.

## Introduction

Low-value agro-industrial by-products as feed for monogastric animals are reasonable to improve resource efficiency and sustainability of livestock farming. However, the use of such by-products is often limited by their poor nutrient digestibility, which is largely explained by a high crude fiber content (Huang and Lee, 2018). One strategy to improve the utilization of these by-products in poultry feed is their valorization by edible fungi, such as *Pleurotus sapidus*, which are capable of degrading a wide range of non-starch polysaccharides and lignin-containing substrates (Valverde et al., 2015). While commercial mushroom products are usually obtained in a time-exhausting process from the fruiting bodies, the submerged cultivation of mushrooms allows a fast and efficient production of mycelial biomass (Papaspyridi et al., 2012). *P. sapidus* mycelia could be of interest for poultry as a source of prebiotic polysaccharides, such as β-glucans, which support intestinal integrity and overall gut and systemic health (Maheshwari et al, 2020).

Considering that knowledge about the use of fungal mycelia in poultry feeding is scarce, we have recently explored the effect of feeding diets with 25 and 50 g/kg diet *P. sapidus* mycelium in broilers (Schäfer et al., 2024). This study demonstrated only marginal effects on the gut microbiome, the liver transcriptome and the plasma metabolome (Schäfer et al., 2024). Despite feed intake and feed:gain ratio did not differ between broilers fed the diets without and with *P. sapidus* mycelium, unpublished data of this study showed a reduction of the relative breast muscle weight in broilers fed the diet with 50 g/kg diet *P. sapidus* mycelium indicating that protein turnover in breast muscle might has been affected.

Protein turnover in skeletal muscle is the result of the activity of protein synthetic and protein catabolic pathways (Ferreira and Duarte, 2023). A key signaling pathway stimulating protein synthesis is the mammalian target of rapamycin (**mTOR**) pathway. In addition, the ribosomal protein S6 kinase (**S6K1**), a down-stream target of mTOR, activates protein synthesis. Furthermore, protein anabolism in skeletal muscle is stimulated by insulin-like growth factor 1 (**IGF1**) and hepatic growth hormone receptor (**GHR**) *via* the somatotropic axis. While the alpha serine/threonine-protein kinase (**AKT**) acts as an upstream activator of the protein anabolic mTOR pathway, AKT negatively regulates the forkhead box protein O1 (**FOXO1**) transcription factor, which stimulates protein degradation. Protein degradation by FOXO1 involves induction of F-box only protein 32 (**FBXO32**) encoding the E3 ligase atrogin-1 mediating protein degradation *via* the ubiquitin-proteasome pathway. The E3 ligase atrogin-1 is also induced by pro-inflammatory stimuli which explains that protein degradation *via* the ubiquitin-proteasome pathway is stimulated during inflammatory conditions. A further protein catabolic pathway is the general control nonderepressible 2 (**GCN2**)/eukaryotic translation initiation factor 2A (**eIF2a**) pathway, which inhibits the mTOR pathway (Métayer et al., 2008), and stimulates protein degradation *via* the autophagy-lysosomal pathway (Finkbeiner, 2020).

Against the abovementioned indications that *P. sapidus* mycelium might has affected protein turnover in breast muscle of broilers, the present study tested the hypothesis that dietary inclusion of *P. sapidus* mycelium modulates the expression of genes involved in protein synthesis and muscle growth as well as protein degradation in breast muscle of broilers.

## Materials and methods

### *Animals and diets*

For this investigation, breast muscle samples from a recently published animal study involving 72 one-day-old broiler chickens (Cobb 500; Cobb-Vantress, Wiedemar, Germany) were utilized (Schäfer et al., 2024). The five-week feeding trial was authorized by the Animal Welfare Officer of the Justus Liebig University Giessen (Approval no.: JLU 843_M). All experimental procedures followed established guidelines for the care and handling of laboratory animals. The broilers (total *N* = 72) were randomly assigned to three different groups (6 cages/group with 4 broilers each) with similar body weights (**BW**), and fed three different nutrient adequate diets containing either 0 (PSA-0), 25 (PSA-25) and 50 (PSA-50) g/kg diet of a biotechnologically produced *P. sapidus* mycelium in a three-phase feeding system (starter diet: day 1-10, grower diet: day 11-21, finisher diet: day 22-35). The composition of the different experimental diets, which had similar concentrations of crude nutrients, sugar, starch, amino acids, fatty acids and metabolizable energy within each feeding phase, and details about the production of *P. sapidus* mycelium *via* submerged fermentation, animal housing conditions, light regime, feeding regime and animal killing procedure can be found in our recent publication (Schäfer et al., 2024).

### *Sample collection*

After slaughtering the animals as recently described (Schäfer et al., 2024), the left and right thigh and the left and right breast muscle were dissected. Thigh and breast muscle weights were obtained from weighing the left and the right part together. For the measurement of expression of genes involved in protein synthesis and protein degradation pathways, small portions of the right breast muscle from 12 broilers per group (2 broilers/cage), whose BW corresponded to the mean of the whole group, were collected, snap-frozen in liquid nitrogen, and stored at –80°C for later analysis.

### *Total RNA extraction and qPCR in breast muscle*

Total RNA extraction from frozen breast muscle portions (40-50 mg) was performed using TRIzol reagent (Invitrogen, Karlsruhe, Germany). The total RNA concentration and the A260/A280 ratio of all samples was 515 ± 46 ng/µL and 1.92 ± 0.02 (total *N* = 36 or *n* = 12/group), respectively. Analysis of RNA quantity and quality, synthesis of cDNA, qPCR analysis using a Rotor-Gene Q system (Qiagen, Hilden, Germany), and calculation of relative mRNA levels with the comparative Ct method using the sample with the lowest Ct value as reference Ct value were carried out as described in a recent publication (Chiappisi et al., 2017). Characteristics of gene-specific primers (Eurofins MWG Operon, Ebersberg, Germany) are available on request from the corresponding author.

### *Western blot analysis in breast muscle*

Approximately 30 mg of frozen breast muscle tissue was homogenized in radioimmunoprecipitation assay buffer containing phosphatase and protease inhibitors. The homogenates were centrifuged, and the supernatants were stored at -20ºC. Following determination of protein concentration of the supernatants, protein separation using SDS-PAGE (10% separating gel, 6% stacking gel) was carried out with different amounts of protein depending on the target antibody [100 µg for phosphorylated and total S6K1 (p-S6K1, S6K1), phosphorylated and total eIF2a (p-eIF2a, eIF2a), phosphorylated and total alpha serine/threonine-protein kinase (p-AKT, AKT); 30 µg for phosphorylated and total ribosomal protein S6 (**p-RPS6**, RPS6)]. In each gel, a molecular mass marker was included (10-170 kDa, PageRuler prestained protein ladder; Thermo Scientific, Dreieich, Germany). Subsequently, the proteins were electro-transferred for 1.5 h at 100 V and 300 mA to a nitrocellulose membrane (Pall, Pensacola, FL, USA) using a blotting buffer with 20% methanol. Ponceau S (Carl Roth, Karlsruhe, Germany) staining was performed to verify equal loading. Afterwards, the membranes were blocked using 5% skimmed milk powder (for p-S6K1, S6K1, p-RPS6 and RPS6) or 5% bovine serum albumin (for p-eIF2a, eIF2a, p-AKT, and AKT) in TBST buffer. The following primary antibodies were used for the detection of p-S6K1, S6K1, p-RPS6, RPS6, p-eIF2a, eIF2a, p-AKT, and AKT: Phospho-p70 S6 Kinase (Thr389) (108D2) rabbit monoclonal antibody (cat. no. 9234), p70 S6 kinase antibody (cat. no. 9202), Phospho-S6 ribosomal protein (Ser235/236) antibody (cat. no. 2211), S6 ribosomal protein (5G10) rabbit monoclonal antibody (cat. no. 2217), Phospho-eIF2α (Ser51) antibody (cat. no. 9721), eIF2α antibody (cat. no. 9722), Phospho-Akt (Ser473) (D9E) XP rabbit monoclonal antibody (cat. no. 4060), Akt (pan) (11E7) rabbit monoclonal antibody (cat. no. 4685; all from Cell Signaling Technology Europe B.V. (CST), Frankfurt, Germany). Vinculin recombinant rabbit monoclonal antibody (42H89L44; Invitrogen) was used as reference antibody. Primary antibodies were diluted 1:1,000 in the blocking buffer and the membranes were incubated with the primary antibody solution overnight at 4°C. After washing, a horseradish peroxidase-conjugated secondary polyclonal goat anti-rabbit IgG antibody (A0545, Sigma-Aldrich, Taufkirchen, Germany) for all primary antibodies was added in 1:10,000 dilution in TBST buffer to the membranes and incubated for 1.5 h at room temperature. After washing, the blots were developed using the ECL Select western blotting detection reagent (GE Healthcare, Munich, Germany) for 5 min at room temperature. The intensities of the specific bands were detected using a Bio-Imaging system (G:Box, Syngene, Cambridge, UK) and quantified using Syngene Gene Tools 4.3.1 software.

### *Statistical analysis*

Statistical analysis was performed using SPSS 28 software (IBM, Armonk, NY, USA). The cage served as the experimental unit for feed intake and feed:gain ratio (*n* = 6 cages/group; published in Schäfer et al. (2024)) and the individual animal for all other data. Because carcass characteristics and breast muscle and thigh weights were obtained for all animals alive at the end of the trail (total animals alive = 65), the means from these data represented *n* = 21-22 broilers/group. Data from qPCR and Western blotting were calculated from a total of 36 animals (*n* = 12 broilers/group), because only 2 broilers/cage (6 cages/group) were used for these analyses. All data were tested for normal distribution using the Kolmogorov-Smirnov test for carcass characteristics and breast muscle and thigh weights and Shapiro-Wilk test for qPCR and Western blotting data. For evaluating homoscedasticity, the Levene's test was used. If the normal distribution was only achieved after log-transformation, the log-transformed data were used for statistical analysis. Differences between the three groups were analyzed using one-way analysis of variance (one-way **ANOVA**) followed by a Tukey's post-hoc test when the data were normally distributed, and the variances were homogeneous. If the variance was heterogenous, the means of the three groups were analyzed using Welch's ANOVA and the Games-Howell post-hoc test. If data were not-normally distributed, a one-way ANOVA according to Kruskal-Wallis was performed using the Mann-Whitney U test with Bonferroni correction as post-hoc test. A *P*-value < 0.05 was considered statistically significant for all analyses.

## Results and discussion

Despite that growth performance (reported in Schäfer et al. (2024)), carcass characteristics, and absolute breast and thigh weights of the broilers was not affected by dietary inclusion of *P. sapidus* mycelium (Table 1), the relative breast muscle weight of broilers was lower in group PSA-50 than in group PSA-0 (*P* < 0.05, Table 1). In order to clarify this effect, we analyzed the expression of several key genes involved in critical pathways regulating protein turnover in breast muscle of these broilers. In addition, we analyzed the phosphorylation of selected proteins, namely S6K1, RPS6, eIF2a and AKT, playing a key role in muscle protein synthesis (Ferreira and Duarte, 2023).

**Table 1:** Carcass characteristics and breast muscle and thigh weights of broilers fed diets with either 0 (PSA-0), 25 (PSA-25) or 50 (PSA-50) g/kg diet *P. sapidus* mycelium for 35 days.

	PSA-0	PSA-25	PSA-50	Pooled SEM	*P*-value
Eviscerated carcass weight, g	2154	2158	2154	93	0.984
Dressing percentage, %	75.1	73.3	73.9	1.5	0.703
Breast muscle, g	674	659	646	34	0.612
Breast muscle, % of BW	23.5^a^	22.4^a^^,^^b^	22.1^b^	0.7	0.044
Thigh, g	574	579	577	26	0.967
Thigh, % of BW	20.0	19.7	19.8	0.5	0.736

Data are means, *n* = 21-22 broilers/group. ^a,b^ Means without a common letter differ across the groups, *P* < 0.05. BW, body weight

Within genes of the mTOR pathway, the mRNA level of *GHR* was higher in group PSA-25 than in group PSA-0, whereas the mRNA level of *IGF1R* was lower in group PSA-25 than in group PSA-0 (*P* < 0.05, Table 2). The mRNA levels of *GHR* and *IGF1R* did not differ between group PSA-50 and the other two groups. Amongst the genes belonging to the mTOR pathway, the mRNA level of *S6K1* was higher in group PSA-25 than in group PSA-0 (*P* < 0.05), whereas the *S6K1* mRNA level was not different between group PSA-50 and groups PSA-25 and PSA-0 (Table 2). The mRNA levels of two other genes of the mTOR pathway, *MTOR* and *4EBP1*, were not different among groups (Table 2). Within the muscle growth-related genes investigated, the mRNA level of *MYOG* was lower in groups PSA-25 and PSA-50 than in group PSA-0 (*P* < 0.05), whereas the mRNA levels of *MYOD1* and *MYF5* did not differ among groups (Table 2). The effect on *MYOG* is noteworthy because MYOG is involved in myotube differentiation (Sabourin and Rudnicki, 2000), which indicates a general inhibitory effect of feeding *P. sapidus* mycelium on muscle cell differentiation of broilers. However, given that the mRNA levels of other muscle growth-related genes in breast muscle were not different between groups, an effect on myotube differentiation is questionable and more research is necessary to clarify this issue.

**Table 2:** Relative mRNA levels of selected genes involved in protein anabolic and protein catabolic pathways in breast muscle of broilers fed diets with either 0 (PSA-0), 25 (PSA-25) or 50 (PSA-50) g/kg diet *P. sapidus* mycelium for 35 days.

	PSA-0	PSA-25	PSA-50	Pooled SEM	*P*-value
	Relative mRNA level, fold of PSA-0				
Somatotropic axis					
*GHR*	1.00^b^	1.62^a^	1.41^ab^	0.28	0.034
*IGF1*	1.00	1.13	0.91	0.25	0.550
*IGF1R*	1.00^a^	0.67^b^	0.81^ab^	0.15	0.024
*IGFBP2*	1.00	0.73	0.80	0.24	0.457
mTOR pathway					
*MTOR*	1.00	1.17	1.21	0.13	0.124
*S6K1*	1.00^b^	1.88^a^	1.67^ab^	0.37	0.019
*4EBP1*	1.00	1.00	1.02	0.19	0.970
Muscle growth					
*MYOD1*	1.00	0.76	0.79	0.18	0.256
*MYF5*	1.00	0.92	1.24	0.21	0.174
*MYOG*	1.00^a^	0.56^b^	0.63^b^	0.15	0.006
GCN/eIF2a pathway					
*SQSTM1*	1.00	1.05	1.00	0.18	0.765
Ubiquitin-proteasome pathway					
*FBXO32*	1.00	1.06	0.78	0.24	0.271
*FOXO1*	1.00	1.13	1.21	0.16	0.241
*MURF1*	1.00	1.21	1.12	0.25	0.587
Autophagy-lysosomal pathway					
*ATG5*	1.00	0.88	0.94	0.16	0.644
*ATG9A*	1.00^b^	1.39^a^	1.26^ab^	0.17	0.033
*BECN1*	1.00	1.26	1.24	0.21	0.246
Inflammation					
*IL1B*	1.00^a^	0.61^b^	1.00^ab^	0.19	0.008
*TLR4*	1.00^a^	0.58^b^	0.77^ab^	0.15	0.007
*TNFA*	1.00^a^	0.58^b^	0.97^ab^	0.26	0.031
*VCAM1*	1.00	0.97	1.17	0.15	0.220

Data are means, *n* = 12 broilers/group. ^a,b^ Means without a common letter differ across the groups, *P* < 0.05.

The relative phosphorylation of S6K1, RPS6, eIF2a and AKT did not differ across the three groups (data not shown). The mRNA levels of most genes involved in pathways of protein degradation and inhibition of protein synthesis, such as the GCN/eIF2a, the ubiquitin-proteasome and the autophagy-lysosomal pathways, showed no differences across the three groups (Table 2). Only the mRNA level of *ATG9A* was higher in group PSA-25 compared to group PSA-0 (*P* < 0.05), but did not differ between group PSA-50 and the other two groups (Table 2). While no effect was seen regarding the mRNA levels of pro-inflammatory genes between groups PSA-50 and PSA-0, the mRNA levels three (*IL1B, TLR4, TNFA*) out of four pro-inflammatory genes were lower in group PSA-25 than in group PSA-0 (*P* < 0.05, Table 2). This anti-inflammatory effect of *P.* spp. mycelia, which has been also documented for rats (Maheshwari et al., 2020), can be considered as beneficial because pro-inflammatory stimuli like IL1β and TNFα promote protein degradation *via* targeting the ubiquitin-proteasome pathway, thereby, impairing protein accretion. Nevertheless, the anti-inflammatory effect seen at the transcriptional level in group PSA-25 obviously had no relevance for the breast muscle gain. Albeit being speculative, one reason might be that the effect was not translated to the protein level keeping the concentrations of pro-inflammatory mediators unaffected.

In overall, considering that only the mRNA level of one muscle growth-related gene (*MYOG*) in breast muscle differed between group PSA-50 and PSA-0, whereas the expression of genes from all other pathways and phosphorylation levels of S6K1, RPS6, eIF2a and AKT did not differ between these two groups, a modulation of these pathways is not very likely to explain the reduced relative breast muscle weight in broilers of group PSA-50. However, we cannot exclude the possibility that feeding 50 g/kg diet *P. sapidus* mycelium affected other genes of these pathways, which were not considered in this study. In addition, it might be possible that feeding *P. sapidus* mycelium affected other less prominent pathways, such as Wnt/β-catenin, Notch and Hippo signaling, which are also involved in the regulation of protein turnover but whose contribution to skeletal muscle mass regulation is less clear (Ferreira and Duarte, 2023). Future studies using genome-wide transcript profiling of breast muscle could help to identify with greater certainty the signaling pathways involved in the regulation of skeletal muscle mass which were affected by feeding *P. sapidus* mycelium.

A rather unexpected finding was that a significant number of genes from the above-mentioned signaling pathways including *IL1B, TLR4, TNFA, GHR, IGF1R, S6K1, MYOG* and *ATG9A* was found to be affected by feeding the low dose of *P. sapidus* mycelium, although relative breast muscle weights did not differ between groups PSA-25 and PSA-0. However, with the exception of pro-inflammatory genes, the regulation of these genes between group PSA-25 and group PSA-0 was very inconsistent. This was evident from the observation that one gene involved in the protein anabolic mTOR pathway (*S6K1*) and one gene involved in the protein catabolic autophagy-lysosomal pathway (*ATG9A*) were simultaneously up-regulated in group PSA-25 compared to group PSA-0. This inconsistency in the effect of feeding 25 g/kg diet *P. sapidus* mycelium was also seen in the somatotropic axis, in which one gene each was up- (*GHR*) and down-regulated (*IGF1R*) in breast muscle of group PSA-25 compared to group PSA-0, whereas relative phosphorylation of proteins involved in protein anabolic pathways in breast muscle did not differ between these two groups (data not shown). Given this inconsistent regulation of genes between group PSA-25 and group PSA-0 and the lack of difference in the relative breast muscle weights between groups PSA-25 and PSA-0, the observed effects on gene expression are obviously not relevant to breast muscle gain. It is not unlikely that the latter is the consequence of the overall weak effects on gene expression as reflected by the less than 2-fold regulation of these genes between groups.

In conclusion, the present findings suggest that a modulation of the protein anabolic or protein catabolic pathways considered in this study is unlikely to explain the reduced relative breast muscle weight in broilers fed 50 g/kg diet *P. sapidus* mycelium. However, it cannot be excluded that feeding *P. sapidus* mycelium affected other less prominent pathways which have not been considered in this study but which are also involved in the regulation of protein turnover.

## Conflict of interest

The authors declare that they have no competing interests.
